# Circulating and urinary microRNAs profile for predicting renal recovery from severe acute kidney injury

**DOI:** 10.1186/s40560-022-00637-0

**Published:** 2022-09-30

**Authors:** Thanawat Phulkerd, Tanat Lertussavavivat, Umaporn Limothai, Sadudee Peerapornratana, Win Kulvichit, Nuttha Lumlertgul, Kriang Tungsanga, Somchai Eiam-Ong, Yingyos Avihingsanon, Nattachai Srisawat

**Affiliations:** 1 Division of Nephrology, Department of Medicine, Faculty of Medicine, Chulalongkorn University and King Chulalongkorn Memorial Hospital, Bangkok, Thailand; 2 Excellence Center for Critical Care Nephrology, King Chulalongkorn Memorial Hospital, Bangkok, Thailand; 3 Center of Excellence in Critical Care Nephrology, Faculty of Medicine, Chulalongkorn University, Bangkok, Thailand; 4 Department of Laboratory Medicine, Faculty of Medicine, Chulalongkorn University, Bangkok, Thailand; 5 Academy of Science, Royal Society of Thailand, Bangkok, Thailand; 6 Tropical Medicine Cluster, Chulalongkorn University, Bangkok, Thailand

**Keywords:** MicroRNAs, Renal recovery, Acute kidney injury, Biomarker

## Abstract

**Background:**

There is little known about the contribution of microRNAs (miRNAs) in the recovery from acute kidney injury (AKI). This study aimed to discover and validate miRNA profiles for predicting renal recovery from severe AKI.

**Patients and methods:**

A prospective observational study was conducted between June 2020 and January 2021. Urine and serum samples of participants with AKI stage 3 were collected from two groups: renal recovery and renal non-recovery. Transcriptomic analysis was performed using nCounter miRNA Expression Assay. Expression levels of candidate miRNAs were validated using quantitative real-time polymerase chain reaction (qRT-PCR).

**Results:**

The discovery phase identified 18 and 11 differentially expressed miRNAs that were statistically significant between the two groups in urine and serum specimens, respectively. Top candidate miRNAs selected included miR-556-3p, miR-1915-3p, miR-4284, miR-32-5p, miR-96-5p, and miR-556-5p in urine, and miR-499b-5p, miR-30a-3p, miR-92b-3p and miR-770-5p in serum. This study enrolled 110 participants in the validation phase. The qRT-PCR analysis indicated that urine miR-556-3p was significantly higher in the renal recovery group than in the renal non-recovery group. Urine miR-556-3p alone predicted renal recovery with an area under the curve (AUC) of 0.64 (95%CI 0.52–0.75, *p* = 0.03). Combining the clinical model with urine miR-556-3p predicted renal recovery with an AUC of 0.83 (95%CI 0.75–0.92, *p* < 0.01).

**Conclusion:**

This data provides evidence that microtranscriptome profiles of severe AKI patients with renal recovery differed from the non-recovery group. Urine miR-556-3p had the potential to improve the prediction of renal recovery from severe AKI.

**Supplementary Information:**

The online version contains supplementary material available at 10.1186/s40560-022-00637-0.

## Introduction

Acute kidney injury (AKI) is a common and significant problem, especially in critically ill patients. It is estimated that between 20 and 50% or more of all ICU patients around the world are reported to have AKI [1]. A more advanced stage of AKI can also put a patient at a higher risk of mortality [2]. AKI is also strongly associated with poorer outcomes including longer hospital stay, renal replacement therapy, and risk of chronic kidney disease (CKD) [3]. Moreover, many studies indicated that the duration of AKI and the pattern of its recovery also affected the prognosis regarding both short- and long-term outcomes [4, 5]. AKI patients in a renal non-recovery group had been found to have a higher hospital mortality rate and higher risk of adverse renal outcomes after hospital discharge [6]. A patient’s recovery course after AKI is often a key determinant of improved long-term outcomes.

In response, the normal physiology of renal recovery after acute insult involves the process of tubular epithelial cells dedifferentiation, proliferation, and redifferentiation [7]. This complex process requires an interaction between many types of cells (e.g., surviving renal tubular epithelial cells, renal specific stem cells, and mesenchymal stem cells) and paracrine (e.g., growth factors). If the kidney sustains a severe injury, maladaptive repair may occur leading to fibrosis, tissue malfunction, and eventually CKD [8, 9].

Predicting renal recovery still relies on traditional biomarkers such as urine output, serum creatinine, and urine NGAL [10]. However, these indicators have limitations because they are slow in response, and hence any intervention meant to rescue kidney function might be delayed [11]. It is important to identify other additional biomarkers that could improve the prediction of renal recovery after AKI. MicroRNAs (miRNAs) are non-coding proteins and small RNA molecules which regulate protein synthesis via post-transcriptional messenger RNA (mRNA) [12]. Several miRNAs have a role in cellular function, including cell cycle arrest, fibrosis, and cell differentiation. They are released from cells and present in serum and urine and are thought to contribute to the recovery process [13, 14].

This study aimed to identify and validate miRNA expressions in urine and serum for predicting renal recovery after severe AKI.

## Materials and methods

### Study design and participants

This prospective observational study was completed at King Chulalongkorn Memorial Hospital, Bangkok, Thailand, between June 2020 and January 2021. Participants aged 18 years and older with AKI stage 3 were enrolled. According to the 2012 KDIGO AKI guideline, stage 3 AKI was defined as serum creatinine (SCr) increased more than 3.0 times baseline SCr or SCr increased more than 4 mg/dl (353.6 µmol/l) or the initiation of renal replacement therapy (RRT) [15]. Eligibility criteria for urine sample were specimen with less than three red blood cells per high power field (RBCs/HPF) or more than three RBCs/HPF, but not seen as dysmorphic RBCs, with less than three white blood cells per high power field (WBCs/HPF). Study exclusions were pregnancy, advanced stage of cancer, single kidney, previous history of RRT within the past 30 days, history of renal transplantation, and CKD, defined as persistent glomerular filtration rate less than 60 mL/min/1.73 m^2^ for at least 3 months.

### Definition

Classification of the cohort was done by comparing SCr at day 28 after diagnosis of AKI with baseline SCr. Baseline SCr was obtained from the lowest SCr within 3 months before enrollment. If past SCr was unobtainable, baseline SCr was estimated from back-calculation with the Modification of Diet in Renal Disease (MDRD) formula [16]. Renal recovery at day 28 was defined as alive, dialysis independent, with SCr levels that returned to within 1.5 times of baseline creatinine. Renal non-recovery at day 28 was defined as SCr level 1.5 times higher than the baseline level or death or dialysis dependence [4].

### Specimen collection

Blood and urine were collected on the first day of AKI diagnosis. A 50-mL specimen of urine was collected and kept at 4 °C for up to 4 h until processing. The urine specimen was centrifuged at 1000 rpm for 10 min to remove cells and debris. Cell-free urine was collected in a new 15–50 mL conical tube and centrifuged at 2500 rpm for 10 min to remove any residual debris or bacterial cells. Cell-free urine was then collected in a new 1.5-mL microcentrifuge tube and stored at – 80 °C for future use. Blood samples of 4 mL were drawn up from clotted blood (serum) and kept at 4 °C for up to 1 h until processing. Blood samples were centrifuged at 3,000 g at room temperature for 10 min. The liquid component (serum) was then drawn into a sterile disposable tube and stored at – 80 °C for future use.

### NanoString nCounter system assays in discovery phase

A total of 10 urine samples (5 each from renal recovery and renal non-recovery group) and 9 serum samples (5 from renal recovery and 4 from renal non-recovery group) were randomly selected to investigate the expression profile of 798 human miRNAs using the nCounter1Human v3 miRNA Expression Assays (NanoString Technologies, Seattle, USA). Approximately 100 ng of total RNA was processed according to the manufacturer’s protocol. All counts were collected and captured by the nCounter Digital Analyzer with 280 fields of view per sample. The miRNA data analysis was performed using the nSolver software. For each miRNA, the raw count data were subtracted from the geometric mean of the negative controls. MiRNA profiling data were normalized using the average signals obtained from the positive controls and the top 100 most highly expressed miRNAs.

### Quantitative real-time polymerase chain reaction (qRT-PCR analysis) in validation phase

Candidate miRNAs in urine and blood with log2 fold-change ≥ 1.5 and *p*-value < 0.05 were selected to validate their circulating expression levels by qRT-PCR analysis. Total RNA was polyadenylated with synthesis stem-loop-poly A. Reverse transcription to cDNA was then performed by RevertAid First Strand cDNA Synthesis Kit (Cat No. 1622, Thermo Scientific, USA). The miRNA levels were quantified from cDNA in duplicate using SYBR Green (Luna Universal qPCR Master Mix, Cat No. M3003, New England Biolabs, Inc., USA) and real-time PCR (StepOnePlus Real-time PCR System, Applied Biosystems, USA) as previously described [17]. The primers for candidate miRNAs used in this work are listed in Additional file 1: Table S1. MiRNA expression levels were normalized to the internal control (hsa-miR16-5p), with the relative expression levels calculated by the 2^−ΔΔCT^ method.

### Outcomes

The primary outcome was identifying miRNAs with predictability in renal recovery at 28 days from severe AKI.

### Statistical analysis

Continuous variables were presented as means ± standard deviation in the case of normal distribution and as a median and interquartile range in the case of non-normally distribution. Student’s *t*-test or Mann–Whitney *U* test was used to analyze the differences between continuous variables. Categorical variables were presented as numbers with percentages and were compared using the Chi-square test. The receiver operating characteristic (ROC) curve analysis was used to evaluate the predictability of miRNAs for renal recovery. Univariate and multivariate logistic regression analyses were used to identify factors associated with renal recovery. We also calculated the IDI (integrated discrimination improvement) and NRI (net reclassification index) [18]. IDI is based on the new model’s ability to improve integrated (average) sensitivity without sacrificing average specificity. As the absolute IDI depends on the event rate of the data, we used the relative IDI to reflect the relative improvement. NRI focuses on reclassification tables constructed separately for subjects with and without events based. A *p*-value of less than 0.05 was considered statistically significant. All statistical analysis was performed with STATA software version 14.2 (StataCorp, Texas, USA), and figures were drawn using GraphPad Prism 8 (GraphPad Software Inc., California, USA).

## Results

### Participant characteristics and outcomes

**Fig. 1:**
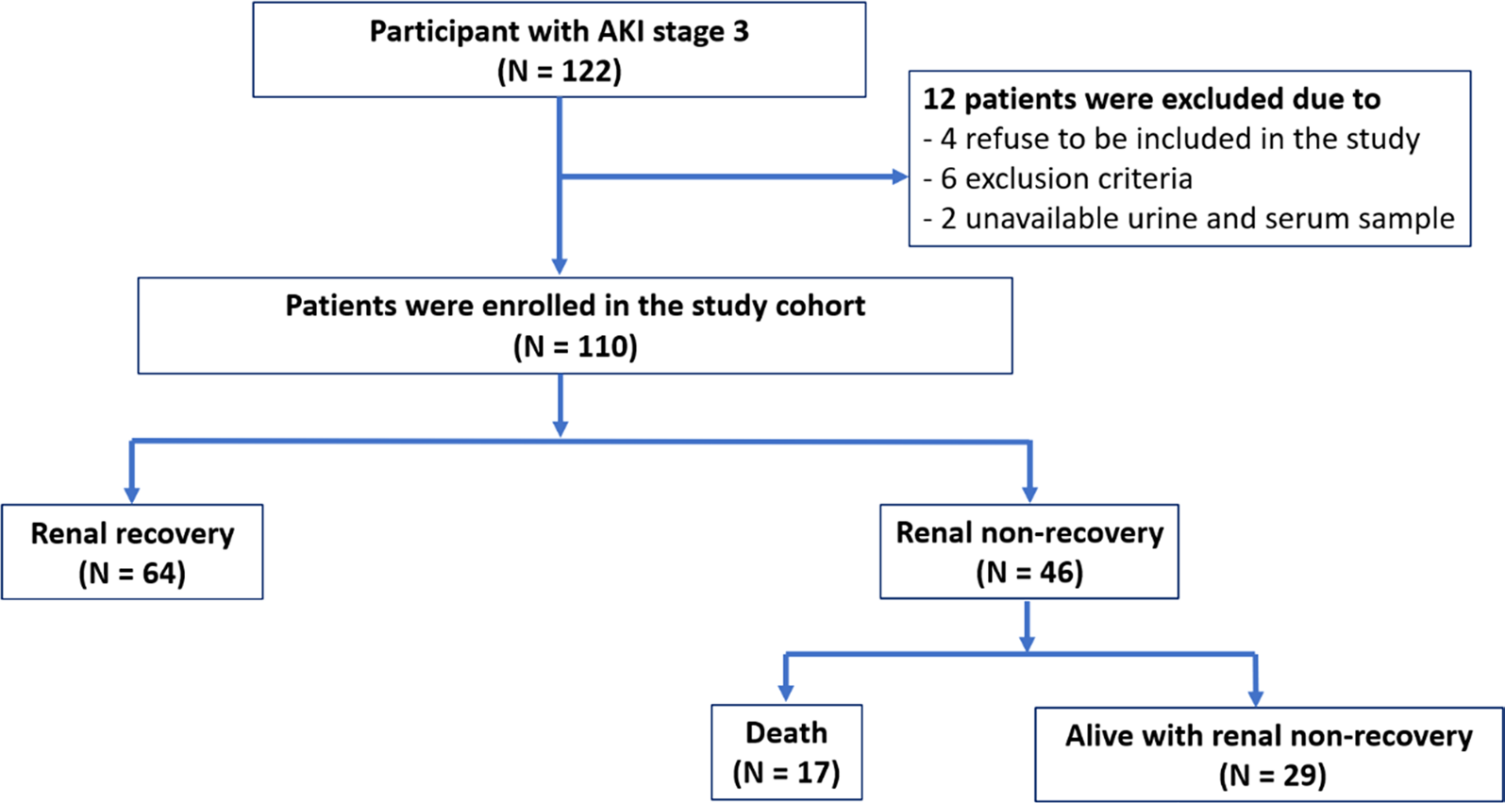
Flow of participants with AKI stage 3 in the cohort study

A total of 122 participants were initially enrolled with AKI stage 3. Six patients who did not meet the inclusion criteria, four patients who refused to participate, and two patients who had inadequate urine and serum samples were excluded (Fig. 1). The final sample for analysis comprised 110 participants with 64 participants achieving renal recovery and 46 participants not achieving renal recovery. The mean age of participants was 60 ± 17 years old. Demographic data of the discovery cohort and validating cohort are presented in Table 1. The study showed several differences in demographic characteristics of the participants in renal recovery group compared to the renal non-recovery group in validating cohort. The renal recovery group had a lower age, less diabetes mellitus and hypertension, lower non-renal Sequential Organ Failure Assessment (SOFA) score, higher mean arterial pressure and higher hematocrit at the diagnosis of AKI than the non-recovery group.

**Table 1:** Baseline demographic data

Clinical characteristics	Discovery phase (Nanostring)			Validation phase (RT-qPCR)		
	Recovery (*N* = 5)	Non-recovery (*N* = 5)	*p* value	Recovery (*N* = 64)	Non-recovery (*N* = 46)	*p* value
Demographic data						
Age; years (mean ± SD)	72.4 ± 9.96	80.2 ± 8.87	0.22	58 ± 17	65 ± 15.68	0.02*
Sex; male (*N*, %)	1 (20)	2 (40)	1	44 (68.75)	26 (56.52)	0.19
Body weight; kg (mean ± SD)	52.1 ± 8.68	55.7 ± 9.05	0.29	61 ± 12.58	59 ± 14.98	0.39
Height; cm (mean ± SD)	157.8 ± 7.33	161.2 ± 6.30	0.45	163 ± 7.73	163 ± 7.19	0.65
BMI; kg/m^2^ (mean ± SD)	26.79 ± 6.51	20.90 ± 7.23	0.21	23.01 ± 4.45	22.27 ± 5.30	0.45
Herb/Nephrotoxic drug (*N*, %)	1 (20)	0	1	4 (6.25)	5 (10.87)	0.38
Comorbid diseases						
Diabetes mellitus (*N*, %)	3 (60)	2 (40)	1	17 (26.56)	21 (45.65)	0.04*
Hypertension (*N*, %)	3 (60)	4 (80)	1	25 (39.06)	29 (63.04)	0.01*
Dyslipidemia (*N*, %)	2 (40)	2 (40)	1	17 (26.56)	20 (43.48)	0.06
Ischemic heart disease (*N*, %)	2 (40)	2 (40)	1	5 (7.81)	9 (19.57)	0.07
Malignancy (*N*, %)	1 (20)	2 (40)	1	5 (7.81)	7 (15.22)	0.22
Cerebrovascular disease (*N*, %)	0	1 (20)	1	6 (9.37)	4 (8.70)	0.9
Smoking (*N*, %)	0	0		2 (3.13)	1 (2.17)	0.76
Main cause of AKI						
Pre-renal/Ischemic ATN, (*N*, %)	0	0		17 (26.56)	15 (32.61)	0.49
Nephrotoxic AKI, (*N*, %)	0	0		5 (7.81)	2 (4.35)	0.46
Contrast induce AKI, (*N*, %)	0	0		0	2 (4.35)	
Septic AKI, (*N*, %)	5 (100)	5 (100)		41 (64.06)	25 (54.35)	0.3
Cast nephropathy, (*N*, %)	0	0		0	1 (2.17)	
Multifactorial, (*N*, %)	0	0		1 (1.56)	1 (2.17)	0.81
Physical examination						
Temperature; °C (mean ± SD)	36.94 ± 0.47	37.44 ± 0.94	0.32	37.11 ± 0.86	37.30 ± 0.73	0.26
SBP; mmHg (mean ± SD)	123.6 ± 10.78	122.4 ± 19.22	0.91	130 ± 20.37	117 ± 19.08	< 0.01*
MAP; mmHg (mean ± SD)	83.8 ± 9.63	86 ± 9.35	0.72	94 ± 13.74	85 ± 13.63	< 0.01*
Laboratory finding						
Hb; g/dL (mean ± SD)	9.42 ± 0.62	9.12 ± 1.76	0.73	9.71 ± 1.88	9.06 ± 1.42	0.05
Hct; % (mean ± SD)	28.12 ± 3.03	27.88 ± 6.54	0.94	29.49 ± 5.65	26.92 ± 4.76	0.02*
WBC; cells × 10^3^ μL (median, IQR)	12.46 (7.01–16.51)	10.02 (8.44–15.49)	0.75	11.65 (8.59–16.28)	12.98 (7.87–17.2)	0.64
Plt; cells × 10^3^ μL (median, IQR)	61 (22–310.5)	247 (123.5–274.5)	0.25	138.5 (66.50–233)	158.15 (79.75–249)	0.17
BUN; mg/dL (median, IQR)	36 (35–47.5)	52 (43.5–99.5)	0.11	56 (42–86)	52 (39–74)	0.27
Baseline Cr; mg/dL (median, IQR)	0.72 (0.70–1.04)	1 (0.80–1.06)	0.12	0.99 (0.80–1.10)	0.93 (0.70–1.20)	0.96
Baseline GFR; mL/min (mean ± SD)	74.85 ± 15.27	64.58 ± 13.03	0.29	82.20 ± 19.01	76.84 ± 27.03	0.22
Admission Cr; mg/dL (median, IQR)	1.4 (0.99–4.65)	1.6 (1.26–4.05)	0.75	3.85 (2.52–4.87)	2.94 (2.29–4.13)	0.57
K; mEq/L (mean ± SD)	3.46 ± 0.34	4 ± 0.70	0.16	3.92 ± 0.85	3.98 ± 0.72	0.74

*ICU* intensive care unit, *SBP* systolic blood pressure, *MAP* mean arterial pressure, *Hb* hemoglobin, *Hct* hematocrit, *Cr* creatinine, *K* potassium, *HCO3* bicarbonate, *Plt* platelets

*p-value < 0.05

The hospital course and outcomes by recovery status are shown in the Additional file 1: Appendix Table S2. The renal recovery group had a lower mechanical ventilation rate (29.69% vs 65.22%, *p* < 0.01), lower use of inotrope drugs (40.63% vs 60.87%, *p* = 0.03) and lower RRT (35.94% vs 71.74%, *p* < 0.01) compared to the non-recovery group. The renal recovery group had a shorter ICU duration (10 vs 26 days, *p* < 0.01), hospital stay (11 vs 23 days, *p* < 0.01) and had lower hospital mortality rate compared to the non-recovery group (1.56% vs 50%, *p* < 0.01).

### Expression profiling of miRNAs in urine and serum

**Fig. 2:**
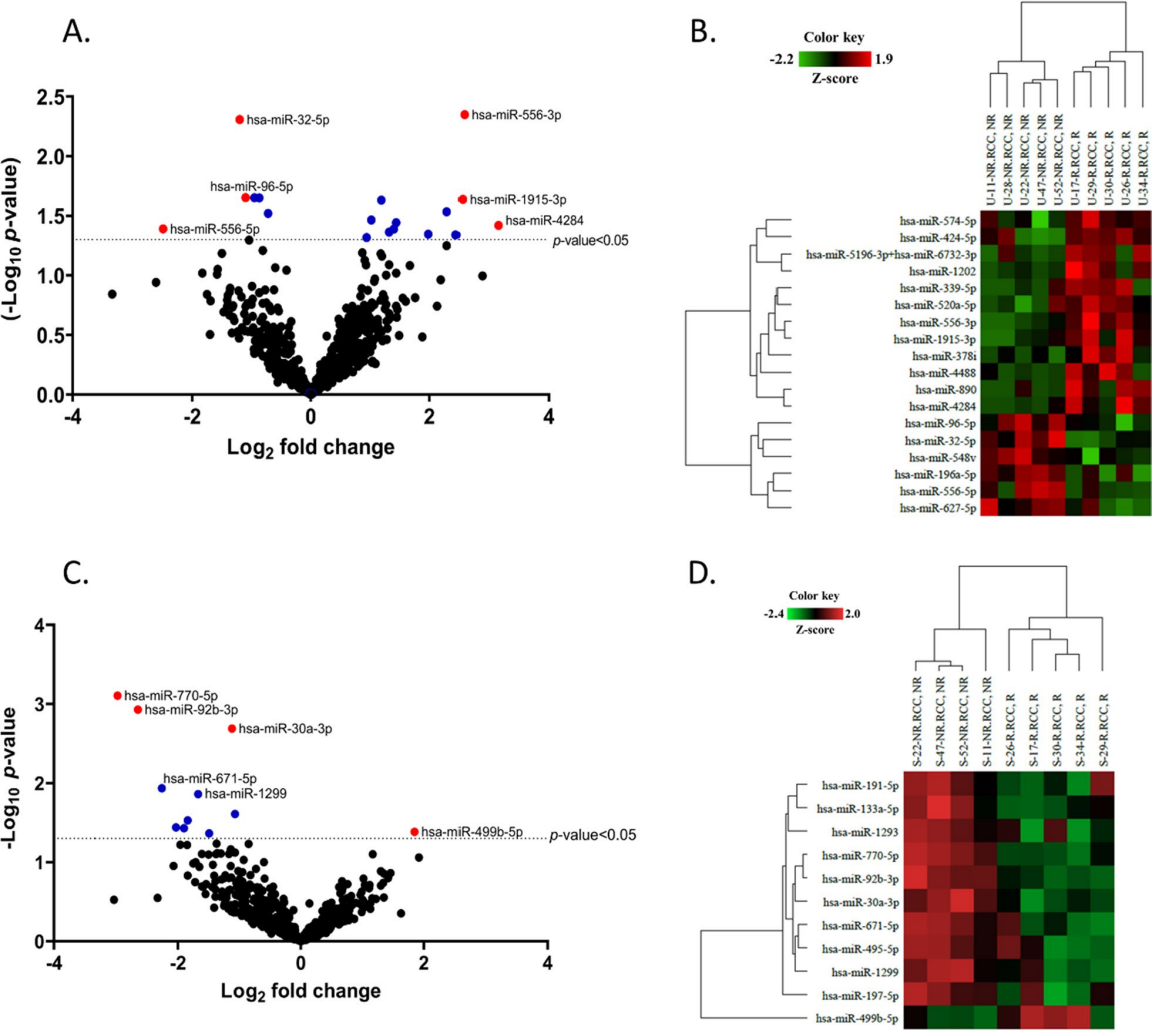
Volcano plot and heatmap for differentially expressed miRNAs between renal recovery group and renal non-recovery group. **A** Volcano plot presents differentially expressed miRNAs in the urine of renal recovery group compared to the non-recovery group. **B **Heatmap of significant differentially expressed miRNAs in the urine of renal recovery group compared to the non-recovery group. **C** Volcano plot presents differentially expressed miRNAs in serum of renal recovery group compared to the non-recovery group. **D** Heatmap of significant differentially expressed miRNAs in serum of renal recovery group compared to the non-recovery group. For the volcano plot, blue scatter dots represent down with *p*-value < 0.05 and red scatter dots represent candidate miRNAs (fold change > 1.5 and *p*-value < 0.05) for qRT-PCR validation

In the discovery phase, 10 samples of urine (five samples each from renal recovery and renal non-recovery group) and 9 samples of serum (four sample from renal recovery group and five samples from renal non-recovery group) were selected for transcriptome analysis of miRNAs. The miRNA expression profiles were obtained from the nCounter system assays. In the urine sample, 18 miRNAs showed significantly different expression between the two groups (Fig. 2A). The hierarchical clustering heatmap of the differentially expressed miRNAs in urine is shown in Fig. 2B. Among these miRNAs, hsa-miR-32-5p, hsa-miR-96-5p, hsa-miR-556-3p, hsa-miR-556-5p, has-miR-1915-3p, hsa-miR-4284 were selected for further qRT-PCR validation.

In the serum sample, 11 miRNAs had significant differential expression between the two groups as illustrated in Fig. 2C. The hierarchical clustering heatmap of the differentially expressed miRNAs in serum is shown in Fig. 2D. Among these miRNAs, hsa-miR-30a-3p, hsa-miR-92b-3p, hsa-miR-499b-5p, hsa-miR-770-5 were selected for further qRT-PCR validation.

### Validation of miRNAs by qRT-PCR

The validated set consisted of 91 urine samples (54 and 37 participants in renal recovery and renal non-recovery group, respectively) and 100 serum samples (54 and 46 participants in renal recovery and renal non-recovery group, respectively). The candidate miRNAs identified in the discovery set were further investigated in both groups of patients.

**Fig. 3:**
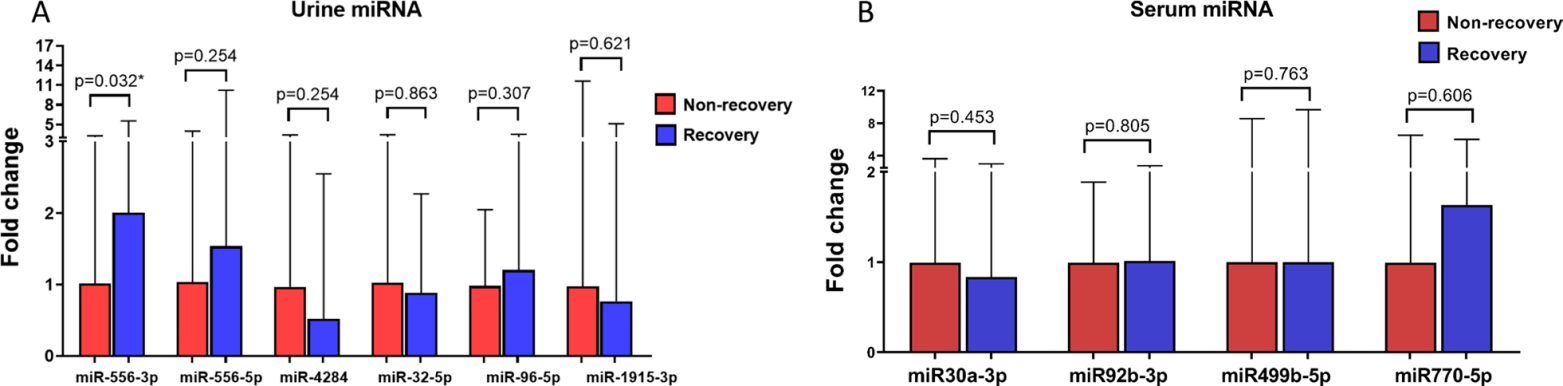
Relative expression of circulating miRNAs between renal recovery group and renal non-recovery group by qRT-PCR analysis. **A** Fold change (relative renal recovery) in urine. **B** Fold change (relative renal recovery) in serum

For urine, the results indicated that the miR556-3p level in the renal recovery group was significantly higher than in the renal non-recovery group (3 [1.12–8.34] vs 1.5 [0.37–4.90], *p* = 0.03). In contrast, there was no significant difference in the level of miR556-5p (1.8 [0.36–12.26] vs 1.2 [0.42–4.79], *p* = 0.25), miR4284 (0.6 [0.09–2.80] vs 1.1 [0.21–3.74], *p* = 0.25), miR32-5p (1.3 [0.32–3.40] vs 1.5 [0.16–5.17], *p* = 0.86), miR96-5p (2.4 [0.43–6.99] vs 2 [0.30–4.10], *p* = 0.31) and miR1915-3p (0.7 [0.14–4.59] vs 0.9 [0.13–10.45], *p* = 0.62). Relative expression urine miRNA levels of the subjects in each group are plotted in Fig. 3A.

For serum, there were no significant difference in the levels of miR30a-3p (0.74 [0.19–2.66] vs 0.89 [0.29–3.23], *p* = 0.45), miR92b-3p (0.96 [0.39–2.62] vs 0.95 [0.49–1.79], *p* = 0.81), miR499b-5p (0.76 [0.17–7.34] vs 0.76 [0.10–6.51], *p* = 0.76) and miR770-5p (1.18 [0.17–4.33] vs 0.72 [0.22–4.69], *p* = 0.61). Relative expression serum miRNA levels of the subjects in each group are plotted in Fig. 3B.

### Prediction of renal recovery

From the result of the validation phase of the study, miR556-3p was selected to be the best candidate for predicting renal recovery. Each cut-off threshold for miR556-3p was used to calculate the sensitivity, specificity, and odds ratio of renal recovery. Table 2 shows the cut-off values of sensitivity and specificity in urine miR556-3p. Urine miR556-3p level cut-off values for prediction of renal recovery were 1.7 (sensitivity 53%, specificity 72%), 2.1 (sensitivity 49%, specificity 75%), and 2.2 (sensitivity 47%, specificity 75%).

**Table 2:** Urine miR556-3p level at various cut-off values for prediction of renal recovery

Cut-off	Sensitivity	Specificity	Odds ratio	95%CI	*p*-value
2.2	0.47	0.75	2.68	1.06–6.77	0.04*
2.1	0.49	0.75	2.89	1.14–7.23	0.03*
1.7	0.53	0.72	2.91	1.18–7.21	0.02*
0.8	0.74	0.44	1.00	0.99–1.00	0.79
0.7	0.77	0.42	1.91	0.73–4.98	0.19
0.6	0.80	0.39	1.91	0.73–4.98	0.19

*CI* confidence interval

*p-value < 0.05

The univariate and multivariate analyses predicting renal recovery are shown in Table 3. The clinical factors significantly associated with renal recovery included non-renal SOFA score, hematocrit, age, and mean arterial pressure. Adjusted clinical factors with urine miR556-3p remained significant (95%CI: 1.28–13.84, *p* = 0.02).

**Table 3:** Analysis of predictors to renal recovery

Characteristic	Univariate analysis		Multivariate analysis	
	Odds ratio (95% CI)	*p*-value	Odds ratio (95% CI)	*p*-value
miRNA 556-3p (cut-off 2.1)	2.89 (1.14–7.23)	0.03*	4.21 (1.28–13.84)	0.02*
DM	2.17 (0.89–5.31)	0.09		
Age (per 5 year)	0.84 (0.72–0.98)	0.04*	0.95 (0.93–0.99)	0.02*
Creatinine on the first day of AKI stage 3	1.01 (0.89–1.15)	0.83		
MAP	1.03 (1.00–1.06)	0.04*	1.04 (1.00–1.08)	0.05
Maximum non-renal SOFA score (per 2 point)	0.62 (0.45–0.84)	< 0.01*	0.77 (0.64–0.92)	< 0.01*
Hct	1.11 (1.02–1.21)	0.02*	1.09 (0.99–1.21)	0.09

*miRNA* microRNA, *DM* diabetes mellitus, *MAP* mean arterial pressure, *SOFA* Sequential Organ Failure Assessment, *Hct* hematocrit

*p-value < 0.05

Prediction of renal recovery with urine miR556-3p alone had an area under the curve (AUC) of 0.64 (95%CI: 0.52–0.75, *p* = 0.03). The AUC of the combined clinical factors including non-renal SOFA score, hematocrit, age, and mean arterial pressure without urine miR556-3p was 0.81 (95%CI: 0.73–0.91, *p* < 0.01). When adding urine miR556-3p into the clinical factors model, AUC was slightly improved to 0.83 (95%CI: 0.75–0.92, *p* < 0.01) (Table 4).

**Table 4:** Stepwise analysis for prediction of renal recovery

Characteristic	Model	AUC (95%CI)	*p*-value
miRNA 556-3p alone		0.64 (0.52–0.75)	0.03
Non-renal SOFA score	A	0.26 (0.16–0.37)	< 0.01
Hematocrit	B	0.65 (0.54–0.76)	0.02
Age	C	0.35 (0.24–0.47)	0.02
MAP	D	0.63 (0.52–0.74)	0.03
A + B		0.75 (0.65–0.85)	< 0.01
A + B + C		0.81 (0.72–0.90)	< 0.01
A + B + D		0.77 (0.67–0.87)	< 0.01
A + B + C + D		0.81 (0.73–0.91)	< 0.01
A + miRNA 556-3p		0.75 (0.65–0.86)	< 0.01
B + miRNA 556-3p		0.69 (0.58–0.79)	< 0.01
C + miRNA 556-3p		0.68 (0.57–0.79)	< 0.01
D + miRNA 556-3p		0.68 (0.56–0.79)	< 0.01
A + B + miRNA 556-3p		0.79 (0.69–0.88)	< 0.01
A + B + C + miRNA 556-3p		0.82 (0.74–0.91)	< 0.01
A + B + D + miRNA 556-3p		0.79 (0.70–0.89)	< 0.01
A + B + C + D + miRNA 556-3p		0.83 (0.75–0.92)	< 0.01

*AUC* area under the curve, *CI* confidence interval, *SOFA* Sequential Organ Failure Assessment, *miRNA* microRNA, *MAP* mean arterial pressure

### Reclassification of renal recovery by miRNA 556-3p

In addition, we tested whether including miRNA 566-3p improved the classification of risk of renal recovery or not. Subjects were categorized into pre-specified high risk, intermediate and low risk of renal non-recovery group and renal recovery group using cut-offs of > 60%, 30–60%, and < 30% based on clinical factors predictive model (Additional file 1: Table S3). With the miRNA 556-3p data added to the clinical factors predictive model, we compared proportions of reclassified subjects across each of these three risk groups. Results showed a significant improvement in the reclassification of the clinical prediction model plus miRNA 556-3p. When miRNA 556-3p was combined with the clinical model (NRI = 44.90%, 95%CI: 0.12–0.88, *p* = 0.01), there was an overall significant improvement in reclassification among recovery and non-recovery subjects. Using the relative IDI, the reclassification of risk of recovery improved by 3.07% when miRNA 556-3p was combined with the clinical model (95%CI: 0.004–0.065, *p* = 0.07).

## Discussion

In the discovery phase, we were able to identify 11 and 18 miRNAs with divergent expression in renal recovery status from serum and urine, respectively. After validated in validation cohorts, we verified significantly increased detection of urinary miR556-3p in patients with renal recovery. We found that only upregulation of miR-556-3p in urine on the first day of AKI stage 3 showed a significant association with renal recovery with an AUC of 0.64. To compare candidate miRNA markers with clinical judgment, we developed a regression model consisting of age, non-renal SOFA score, hematocrit, and mean arterial pressure. Adding urine miR556-3p into the clinical factors model, AUC was slightly improved to 0.83.

Even though the AUC was scant in single miRNA marker and may not provide direct clinical benefit by itself, this finding encouraged further studies to use the miRNA markers for predicting renal recovery. Improving the predictability of miRNA marker could be achieved by combining multiple markers, severity selection, and comparing change over time. Combining clinical factors with miRNA biomarkers is reasonable since multiple clinical factors influence the outcome and should be taken into account.

In addition, miRNAs profiles in serum could not show any significant difference in expression between renal recovery and non-renal recovery. From this study, we found significance only in urine which may be due to the higher correlation between miRNAs in urine and kidney-borne miRNAs than in blood, indicating that urine miRNA is specific to kidney-related diseases.

The function of microRNAs in recovery after AKI has been suggested. Collino et al. evaluated the effect of the global suppression of miRNA biogenesis by the generation of Drosha-knockdown mesenchymal stromal cells (MSCs) [19]. Drosha is an enzyme cleaving the inactive pri-miRNA into the precursor miRNA [20]. With Drosha depleted, MSCs released extracellular vesicles with deregulated miRNA contents. The result showed that the downregulation of miRNAs in MSCs and their extracellular vesicles attenuated their regenerative properties in vivo. In AKI, miRNAs transferred to injured proximal tubular epithelial cells via extracellular vesicles possibly regulated genes involving apoptosis, cytoskeleton reorganization, epithelial–mesenchymal transition, and fibrotic process [21].

The role of miRNA-556-3p in renal recovery is yet unclear. In the database of miRDB, miRBase, and TargetScan Human, miR-556-3p targets many genes including DAB2IP (DAB2 interacting protein) gene [22]. DAB2IP is one of the Ras GTPase-activating protein family and has many functions including regulating cell proliferation and epithelial–mesenchymal transition (EMT) [23]. EMT is one of several processes of dedifferentiation during renal tubular epithelial cells regeneration, described as the process by which damaged cells acquire mesenchymal properties and transform into fibroblasts [24]. Whether EMT transition also plays a role in the process of kidney fibrosis is still debatable [25]. Overexpression of miRNA-556-3p resulted in decreased DAB2IP expression and increased cell proliferation via the Ras–ERK pathway [26]. Furthermore, miR-556-3p was also reported to target the Calpain gene which encodes calcium-dependent cysteine proteases in mammals [22]. Calpains activation promotes endothelial cell apoptosis by its degradative activity and increases endothelial permeability [27]. Rodent study showed reduced LPS-induced renal dysfunction in endothelial cell-specific Capn4 knockout mice [28]. Gene regulation through DAB2IP and Calpain may be one of several mechanisms by which miRNA-556-3p affects renal recovery. We did not evaluate the expression of DAB2IP or Calpain in this study and hence could not confirm or deny the proposed underlying mechanism. Further research regarding the roles of miRNA in the complex renal recovery process could provide the better understanding of the function for discovered miRNA markers.

Other significant miRNAs in urine found in the discovery phase did not show significance in the validation phase but some were reported to have a pathophysiologic association with AKI recovery. For example, overexpression of the miR-32-5p inhibited autophagy and promoted human kidney proximal tubular epithelial fibrosis, inflammation, and EMT via targeting mothers against decapentaplegic homolog 7 (SMAD7) [29]. Allograft tissues study showed higher miR-32 expression in kidney tissue during chronic allograft dysfunction with interstitial fibrosis and tubular atrophy, suggesting that miR-32 may be linked to renal fibrosis [30].

In addition to the miRNAs in our study, there were other miRNAs from previous research that can predict renal recovery. Lucy et al. used urinary miRNA profiling to identify miRNA with the potential ability to predict renal recovery at 90 days after severe AKI and reported significant differences in urinary miR-141 and miR-192 expression between recovery/non-recovery groups [31]. ROC curve analysis of renal recovery predictability was 0.63 for miR-141, 0.76 for miR-192, and 0.83 for a combination of both. Comparing predictability with clinical factors was not done in this study, but the underlying mechanism of miR-141 in AKI and renal recovery was proposed and extensively discussed. In short, miR-141 expression was associated with AKI non-recovery, believing it repressed protein tyrosine phosphatase receptor type G (PTPRG) expression and increased proximal tubular epithelial cells death.

Our study has several strengths. First, we used the NanoString nCounter gene expression system to discover the candidate miRNAs. This technique requires only a small number of samples, but offers comprehensive quantitative miRNA profiles with high sensitivity and specificity. Second, we have chosen to test both blood and urine to minimize bias from (1) severe oliguria which is common in patients with severe AKI and may preclude the availability of urine, and (2) potentially confounding alterations in urine biomarker concentrations that can be induced by volume status and diuretic therapy. Lastly, we were able to obtain the complete patient data, follow-up data, and a clear definition of renal recovery. While measuring miRNA requires specialized laboratory and is not generally available in real practice, this research advanced the roles of miRNA in predicting renal recovery where practical marker still lacks. For example, one could identify potential proteins that were regulated by these miRNAs to develop biomarkers that correlate with the physiology of renal recovery and are easily measured with an enzymatic method.

We identified several limitations in the study. First, the urine and serum samples were tested only on the first day of AKI stage 3. This limited our ability to test the predictive value of miRNAs at other time points and the relationship between the change in miRNAs and renal recovery. The interval between the actual onset of AKI and specimen collection might also affect the volume of miRNA expression owing to a different phase of AKI. The five-phase model of renal injury has been described (prerenal, initiation, exacerbation, maintenance, and recovery) and molecular responses to injury in each phase are altered [32]. Nevertheless, risk stratification and prognostication using a biomarker are likely to be useful only when biomarker concentrations are measured early. Second, the specificity of miRNAs for distinctive condition is limited and easily confounded by the complex pathological process of disease including AKI. The cause of AKI, the process of care variables, and comorbidities could affect the miRNA expression and AKI recovery. For instance, we were unable to assess the influence of fluid resuscitation on miRNA expression and renal recovery. Furthermore, different techniques for miRNA isolating, storing and quantifying could also interfere with the result. In our study, we used commercial technology which is standardized, reducing the technique variation between tests. Third, the severity of disease in the recovery/non-recovery group was substantially different regarding comorbidities, need for interventions, length of stay, and in-hospital mortality. This was not unexpected since fragile patients were more likely to develop adverse outcomes simultaneously. Lastly, we did not compare miRNA markers to available markers such as urinary NGAL or TIMP2-IGFBP7. Since miRNAs regulate the RNA translation, miRNA could be an earlier predictor compared to protein markers.

## Conclusion

In conclusion, we suggest that the post-transcriptional regulation of miRNA expression may play an important role in the prediction of renal recovery. Our study showed that the miR-556-3p in urine had the potential to improve the prediction of renal recovery from severe AKI.

## Supplementary Information

**Additional file 1. Supplementary appendix. Table S1. **Oligonucleotide primers used in this study. **Table S2. **Hospital course and outcomes by renal recovery. Risk reclassification using miR556-3p and clinical predictors compared with clinical predictors alone. **Figure S1.** The area under the curve (AUC) for prediction of renal recovery. **Figure S2. **Study design in virtual abstract.

## Data Availability

The datasets used and/or analyzed during the current study are available from the corresponding author on reasonable request.

## Abbreviations

AKI: Acute kidney injury
AUC: Area under the curve
CI: Confidence interval
CKD: Chronic kidney injury
IDI: Integrated discrimination improvement
miRNA: MicroRNA
MSC: Mesenchymal stromal cells
NRI: Net reclassification index
ROC: Receiver operating characteristic
RRT: Renal replacement therapy
SCr: Serum creatinine
SOFA: Sequential Organ Failure Assessment
