# Supplementary material for: Circulating and urinary microRNAs profile for predicting renal recovery from severe acute kidney injury
Source: J Intensive Care. 2022 Sep 30;10:45. doi: 10.1186/s40560-022-00637-0 (PMC9523985; doi:10.1186/s40560-022-00637-0)
Supplement: Supplementary file 1 — Additional file 1. Supplementary appendix. Table S1. Oligonucleotide primers used in this study. Table S2. Hospital course and outcomes by renal recovery. Risk reclassification using miR556-3p and clinical predictors compared with clinical predictors alone. Figure S1. The area under the curve (AUC) for prediction of renal recovery. Figure S2. Study design in virtual abstract. [file 40560_2022_637_MOESM1_ESM.docx]

**Supplementary appendix**

**Table S1. Oligonucleotide primers used in this study**

| **No** | **Primer Name** | **Primer Sequence** |
| --- | --- | --- |
| 1 | miRNA-universal reverse primer | 5'-GCAGGGTCCGAGGTATTC-3' |
| 2 | hsa-miR-499b-5p | 5'-ACAGACTTGCTGTGATGTTCA-3' |
| 3 | hsa-miR-4284 | 5'-GGGCTCACATCACCCCAT-3' |
| 4 | hsa-miR-556-3p | 5'-ATATTACCATTAGCTCATCTTT-3' |
| 5 | hsa-miR-30a-3p | 5'-CTTTCAGTCGGATGTTTGC-3' |
| 6 | hsa-miR-92b-3p | 5'-TATTGCACTCGTCCCGGC-3' |
| 7 | hsa-miR-770-5p | 5'-TACCACGTGTCAGGGCCA-3' |
| 8 | hsa-miR-1915-3p | 5'-CCAGGGCGACGCGGCGGGA-3' |
| 9 | hsa-miR-96-5p | 5'-TTTGGCACTAGCACATTTTTGC-3' |
| 10 | hsa-miR-32-5p | 5'-ATTGCACATTACTAAGTTGC-3' |
| 11 | hsa-miR-556-5p | 5'-GATGAGCTCATTGTAATATGAGA-3' |
| 11 | hsa-miR-16-5p | 5'-CAGCACGTAAATATTGGCG-3' |
| 14 | stem-loop-poly A | 5'-GTCGTATCCAGTGCAGGGTCCGAGGTATTCGCACTGGATACGA  CAAAAAAAAAAAAAAAAAAVN-3' |

**miRNA extraction**

For urine, total RNA was extracted from 5 mL of urine samples using the Urine Exosome RNA Isolation Kit (Cat 47200, Norgen biotek, Canada) according to the manufacturer’s protocol. For serum, total RNA was extracted from 200 µL of serum using the miRNeasy Serum/Plasma Kit (Qiagen, Gaithersburg, MD, USA) according to the manufacturer’s protocol. C. elegans synthetic miR-39 mimic were spiked into each serum samples as an internal control (Cat. 219610, Qiagen, USA). The RNA concentration and purity were measured using the NanoDrop 2000 spectrophotometers (Thermo Scientific, USA).

**Table S2. Hospital course and outcomes by renal recovery**

| **Characteristric** | **No (%)** | | ***P*-value** |
| --- | --- | --- | --- |
|  | **Recovery (n=64)** | **Non-recovery (n=46)** |  |
| Mechanical ventilation (n, %) | 19 (29.69) | 30 (65.22) | <0.01 |
| Inotrope (n, %) | 26 (40.63) | 28 (60.87) | 0.03 |
| Received renal replacement therapy in the hospital (n, %) | 23 (35.94) | 33 (71.74) | <0.01 |
| Duration of ICU (SD) | 10 (8.75) | 26 (22.83) | <0.01 |
| Length of hospital stay; days (IQR) | 11 (7, 20) | 23 (14, 45) | <0.01 |
| Hospital mortality, n (%) | 1 (1.56)* | 23 (50) | <0.01 |

* 1 death in recovery group occurred after 28 days from AKI diagnosis

Abbreviations: ICU, intensive care unit.

**Table S3. Risk reclassification using miR556-3p and clinical predictors compared with clinical predictors alone**

| **Model with clinical predictors alone^a^** | **Model with miR556-3p and clinical predictors** | | | **Direction of reclassification** | |
| --- | --- | --- | --- | --- | --- |
|  | **< 30% risk** | **30-60% risk** | **>60% risk** | **Increase risk** | **Decrease risk** |
| **Renal recovery (N=53)** |  |  |  |  |  |
| < 30% risk | 3 | 0 | 0 | 1 (1.89) | 2 (3.77) |
| 30-60% risk | 1 | 6 | 2 |  |  |
| >60% risk | 0 | 3 | 38 |  |  |
| **Renal non-recovery (N=36)** |  |  |  |  |  |
| <30% | 10 | 1 | 0 | 4 (11.11) | 6 (16.67) |
| 30-60% risk | 1 | 14 | 2 |  |  |
| >60% risk | 0 | 1 | 7 |  |  |

^a^ Clinical predictors include non-renal SOFA score, hematocrit, age, and mean arterial pressure

**Figure S1.** **The area under the curve (AUC) for prediction of renal recovery**


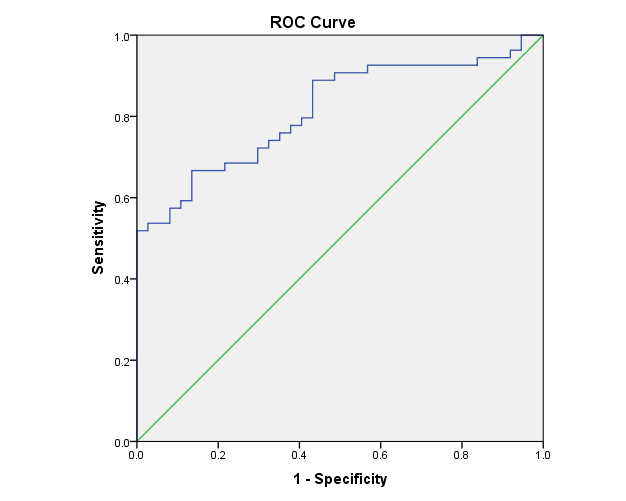


AUC 0.81, p <0.01


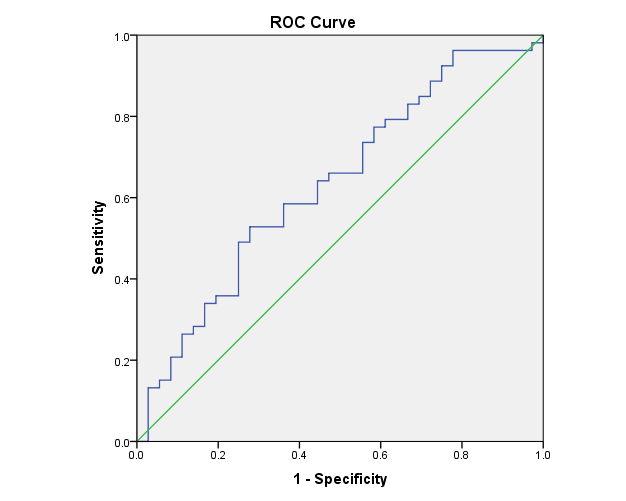


AUC 0.64, p = 0.03

(A) miRNA 556-3p (B) non-renal SOFA score + Hct + Age


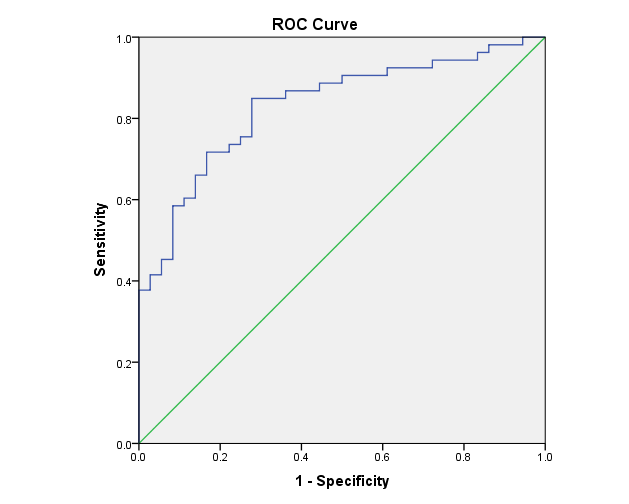


AUC 0.83, p <0.01


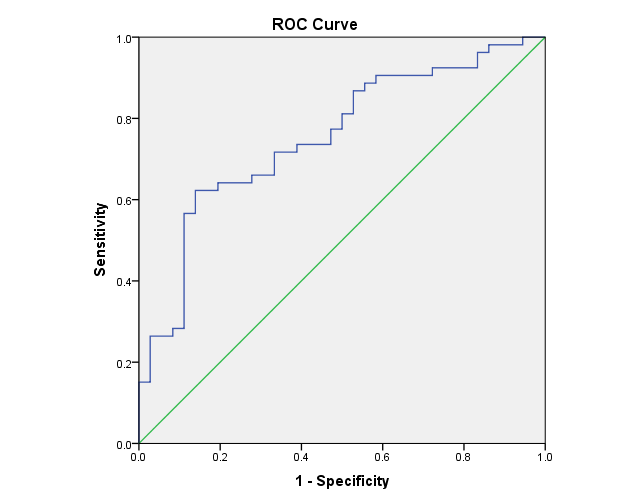


AUC 0.75, p <0.01

(C) miRNA 566-3p + non-renal SOFA score (D) miRNA566-3p + non-renal SOFA score + Hct + Age + MAP

Abbreviations: Hct, hematocrit; SOFA, sequential organ failure assessment; miRNA, microRNA; MAP, mean arterial pressure.

* *P*-value < 0.05


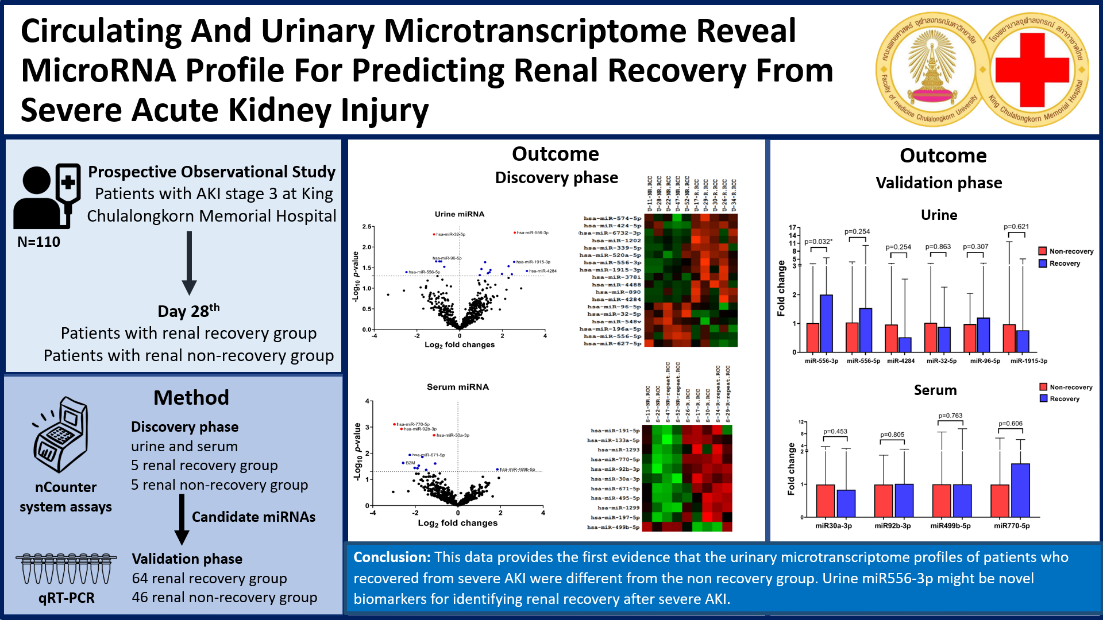
**Figure S2. Study design in virtual abstract**
